# Asian American Diversity and Representation in the Health Care Workforce, 2007 to 2022

**DOI:** 10.1001/jamanetworkopen.2024.40071

**Published:** 2024-10-17

**Authors:** Michelle Ko, Kevin Dinh, Sarah Iv, Monica Hahn

**Affiliations:** 1 Division of Health Policy and Management, Department of Public Health Sciences, University of California, Davis; 2 Department of Economics, University of California, Davis; 3 Office of Health Equity, Diversity, and Inclusion, University of California Davis Health; 4 Department of Family and Community Medicine, School of Medicine, University of California, San Francisco

## Abstract

**Importance:**

Asian American individuals comprise over 40 ethnoracial groups but are regularly aggregated into 1 category within health workforce analysis, thus obscuring substantial inequities in representation.

**Objectives:**

To describe trends in Asian American diversity across the 4 most populous US health professions (physicians, registered nurses, nursing assistants, and home health aides) and to characterize subgroup representation within professions.

**Design, Setting, and Participants:**

Serial cross-sectional study from American Community Survey (ACS) population estimates of people reporting health profession occupations from 2007 to 2022. The ACS samples US residents every day, with approximately 3.5 million surveyed each year. Based on their sampling methods, the ACS then produces estimates of the entire US population for 1-year, 3-year, and 5-year periods. Data were analyzed from April to August 2024.

**Main Outcomes and Measures:**

For each year and subgroup, the proportions of the US population, the profession, and Asian American individuals within the profession were calculated. The representation quotient (RQ) of each subgroup was then calculated, defined as the proportion of the subgroup within the profession of interest divided by the proportion of the subgroup within the US population. Trends were examined over the 15-year period.

**Results:**

Over the 15-year period, Indian Americans composed the largest percentage of Asian American physicians (mean [SD], 40.6% [1.6%]), followed by Chinese Americans (mean [SD], 18.9% [1.4%]). Pakistani and Indian Americans had the highest relative representation (mean [SD] RQ, 8.9 [0.9] and 7.8 [0.9], respectively). Conversely, Cambodian and Hmong Americans remained largely underrepresented (mean [SD] RQ, 0.2 [0.2] for both). Filipinx Americans accounted for more than half of Asian American registered nurses and nursing assistants, with high relative representation (mean [SD] RQ, 5.6 (0.3) and 2.9 [0.4], respectively). Bangladeshi and Chinese American relative representation were high among home health aides (mean [SD] RQ, 4.1 [1.5] and 2.7 [0.5], respectively). Asian American individuals accounted for an estimated 22% of physicians (approximately 260 693 respondents), 10% of registered nurses (approximately 420 418 respondents), 4.8% of nursing assistants (approximately 93 913 respondents), and 8.3% of home health aides (approximately 60 968 respondents) in 2022.

**Conclusions and Relevance:**

By examining disaggregated data, this study found persistent inequities among Asian American subgroups in the health workforce. Reducing Asian American populations to a single racialized group erases subgroup differences rooted in histories of racism, colonialism, and xenophobia; enables false narratives of Asian American overrepresentation and success; and hampers progress in advancing health justice.

## Key Points

**Question:**

Which Asian American subgroups have been represented in major US health care professions in recent years?

**Findings:**

In this serial cross-sectional study of American Community Survey 1-year estimates from 2007 to 2022, Filipinx Americans had consistently high representation among registered nurses and nursing assistants; Indian, Pakistani, and Chinese Americans predominated among physicians, whereas Hmong and Cambodian American representation in medicine remained substantially below general population representation. Bangladeshi and Chinese American representation grew among home health aides over the study period.

**Meaning:**

These findings suggest that aggregation of Asian American subgroups into a single racialized group erases substantial inequities in health workforce diversity.

## Teaser

This serial cross-sectional study evaluates trends in Asian American diversity among physicians, registered nurses, nursing assistants, and home health aides to characterize subgroup representation within professions.

## Introduction

Asian American people are the fastest growing group in the US, composed of 23 million individuals from over 40 ethnoracialized subgroups.^1^ Asian American individuals have the highest socioeconomic inequality of any major racialized group, from Burmese (median household income, $44 000) to Indian Americans ($119 000)^2^; only 21% of Cambodian Americans hold bachelor’s degrees, compared with 57% of Chinese Americans.^3^ The characterization of Asian American people as a monolithic group obscures the underlying diversity of this community and reinforces the model minority myth, the false stereotype of Asian American people as educationally and economically successful due to superior work ethics and prioritization of educational attainment.^4^

Despite the heterogeneity of the population, health workforce studies continue to measure Asian America in aggregate, a form of structural racism that erases disparate communities^5^ and perpetuates a false narrative of Asian American overrepresentation in the health professions. Asian American people have long called for data disaggregation to counteract the practice of reducing a multitude of communities into a single, logic-defying category.^6^ For example, Asian American individuals make up over 20% of medical students, trainees, and physicians, far exceeding their representation in the US population (7%).^7,8^ Based on this relative representation in medicine, discussions of health workforce diversity typically characterize Asian American populations as not underrepresented, overrepresented, or exclude Asian American people entirely.^7,8^ Only by disaggregation can we understand whether the health workforce reflects or even reinforces these same socioeconomic and racialized inequities. The last report of Asian American diversity in medicine—published 10 years ago—found that Chinese, Indian, and Pakistani Americans made up the majority of Asian American physicians and were overrepresented, whereas Filipinx Americans were underrepresented.^9^

Furthermore, the focus on the medical profession fuels existing model minority stereotypes. This practice ignores Asian American representation across a range of health professions, from direct care workers to high-level nursing positions. Examination of multiple professions is particularly salient as major contemporary paths of Asian immigration to the US include direct recruitment of health care workers. Representation itself should not be considered an unqualified good, as occupational segregation within health care itself may contribute to Asian American socioeconomic inequities,^2^ as well as income-related mortality inequities among health care workers.^10^

Health profession education and workforce research needs an updated and expanded discussion. In this study, we describe trends of Asian American subgroup representation in the US population over 15 years and among the 4 most populous health professions. We argue that the default assumption, that Asian American populations are overrepresented, requires greater scrutiny, and the characterization of Asian American people as a single racialized group limits our understanding of diversity, equity, and inclusion in the health professions.

## Methods

### Design, Setting and Data Sources

We conducted a serial cross-sectional study using American Community Survey (ACS) 1-year estimates from 2007 to 2022 to describe annual trends in Asian subgroup representation in the US population and the following 4 self-reported occupations: physician, registered nurse (RN), nursing assistant (NA), and home health aide (HHA). We highlighted these professions to represent both the most populous health workforce occupations, as well as a broad range of professional and socioeconomic strata.

The study procedures and results are reported in accordance with Strengthening the Reporting of Observational Studies in Epidemiology (STROBE) reporting guidelines. The study protocol was reviewed by the UC Davis institutional review board and was determined to be exempt from the need for approval and for informed consent because the study did not involve human participants and used deidentified data.

### Participants

We defined Asian American as “individuals with origins in any of the original peoples of Central or East Asia, Southeast Asia, or South Asia,” consistent with the updated US Office of Management and Budget guidelines.^11^ We excluded Pacific Islanders and Native Hawaiians, who have been officially recognized as distinct groups by the federal Office of Management and Budget since 1997. We classified those who reported 2 or more races, including at least 1 Asian American, as Other Asian (see eAppendix in Supplement 1 for survey items). Due to limitations in the ACS data, we were unable to include major Central and Western Asian subgroups, such as Afghan Americans.

### Statistical Analysis

For each year and subgroup, we calculated each subgroup’s annual proportion of the US population, of each profession, and of Asian American individuals within the profession. We calculated the proportion of each subgroup of all Asian American individuals within each profession to inform potential differential representation related to historical and immigration policy contexts, as well as emphasize the extent of subgroup predominance or erasure within that profession.

Next, we quantified the relative representativeness of each group within each profession for each year using the RQ value. The RQ value is defined as the proportion of the subgroup within the profession of interest, divided by the proportion of the subgroup within the US population.^12^ An RQ greater than 1 indicates higher proportionate representation in the profession vs population, and the converse for RQs less than 1. We calculated the mean and SD of the RQ across all years. We then examined trends in RQ for Asian American individuals in aggregate for each profession, then for each subgroup within each profession using simple linear regressions of year on RQ. We used Stata version 17SE (StataCorp LLC) with extension package grc1leg for analyses. Data were analyzed from April to August 2024.

## Results

**Figure 1.**
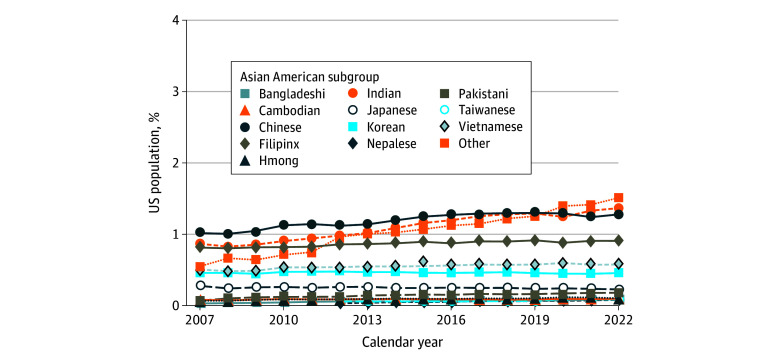
Major Asian American Subgroups by Percentage of US Population, 2007 to 2022 Before 2012, the American Community Survey did not provide separate estimates for residents who identified as Nepalese or Taiwanese American. Estimates of Taiwanese Americans remain in flux and may contribute to small shifts in estimates of Chinese Americans.^40^

In 2022, Asian American individuals made up an estimated 7% (approximately 23 663 384 individuals) of the US population, and the largest subgroups were Other Asian (approximately 5 025 211 individuals [1.5%]), Indian (approximately 4 535 371 individuals [1.3%]), Chinese (approximately 4 243 627 individuals [1.3%]), Filipinx (approximately 3 000 067 individuals [0.9%]), and Vietnamese (approximately 1 893 757 individuals [0.6%]) Americans (Figure 1). We present findings from the 13 most populous subgroups in 2022 (see eTable 1, eTable 2, eTable 3, eTable 4, and eTable 5 in Supplement 1 for analyses for all Asian subgroups identified in ACS). Within the health professions, Asian American individuals comprised an estimated 22% of physicians (approximately 260 693 respondents), 10% of RNs (approximately 420 418 respondents), 4.8% of nursing assistants (NAs) (approximately 93 913 respondents), and 8.3% of home health aides (HHAs) (approximately 60 968 respondents) in 2022. Before 2018, the ACS combined NAs and HHAs into 1 category, so we only present separate estimates from 2018 to 2022.

**Figure 2.**
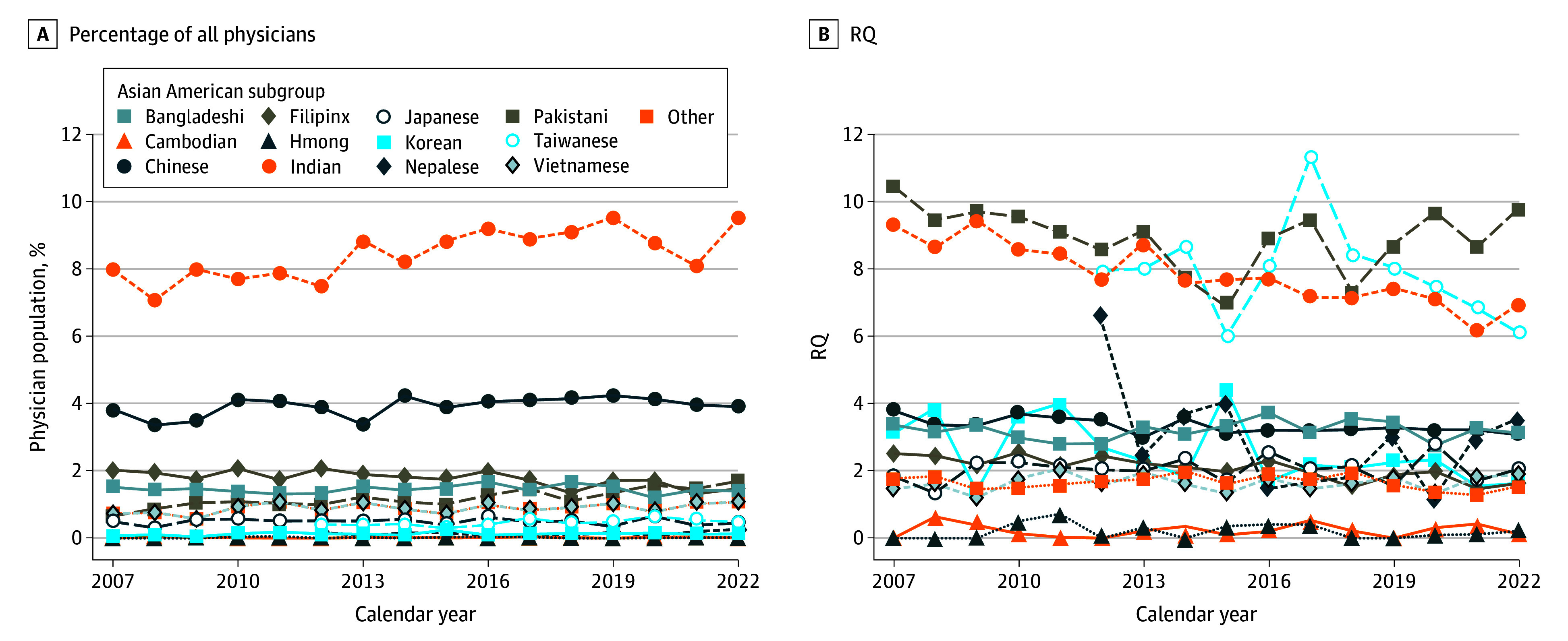
Major Asian American Subgroup Representation Among Physicians, 2007 to 2022 A, Major Asian American subgroups as a percentage of all US physicians; B, major Asian American subgroup representation among physicians, relative to their representation in the US population. RQ indicates representation quotient.

Indian Americans composed the largest percentage of Asian American physicians (mean [SD], 40.6% [1.6%]), followed by Chinese Americans (mean [SD], 18.9% [1.4%]). Among physicians, Indian and Chinese Americans were the 2 largest subgroups, at nearly 13% of all US physicians and more than 50% of all Asian American physicians over time (US physicians annual mean [SD], 989 558 [80 275]; Asian American physicians annual mean [SD], 206 844 [32 546]) (Figure 2A). Indian and Pakistani populations had the highest relative representation (mean [SD] RQ, 7.8 [0.9] and 8.9 [0.9], respectively) (see eTable 1 in Supplement 1 for all subgroups). For Cambodian and Hmong Americans, relative representation increased over time but at a small magnitude (Cambodian: coefficient, 0.03; 95% CI, 0.01 to 0.05; Hmong: coefficient, 0.04; 95% CI, 0.03 to 0.06). Relative representation of Indian American physicians declined over the 15-year period (coefficient for linear trend, −0.17; 95% CI, −0.22 to −0.13); no subgroup exhibited increasing relative representation (see eTable 1 in Supplement 1 for detail).

**Figure 3.**
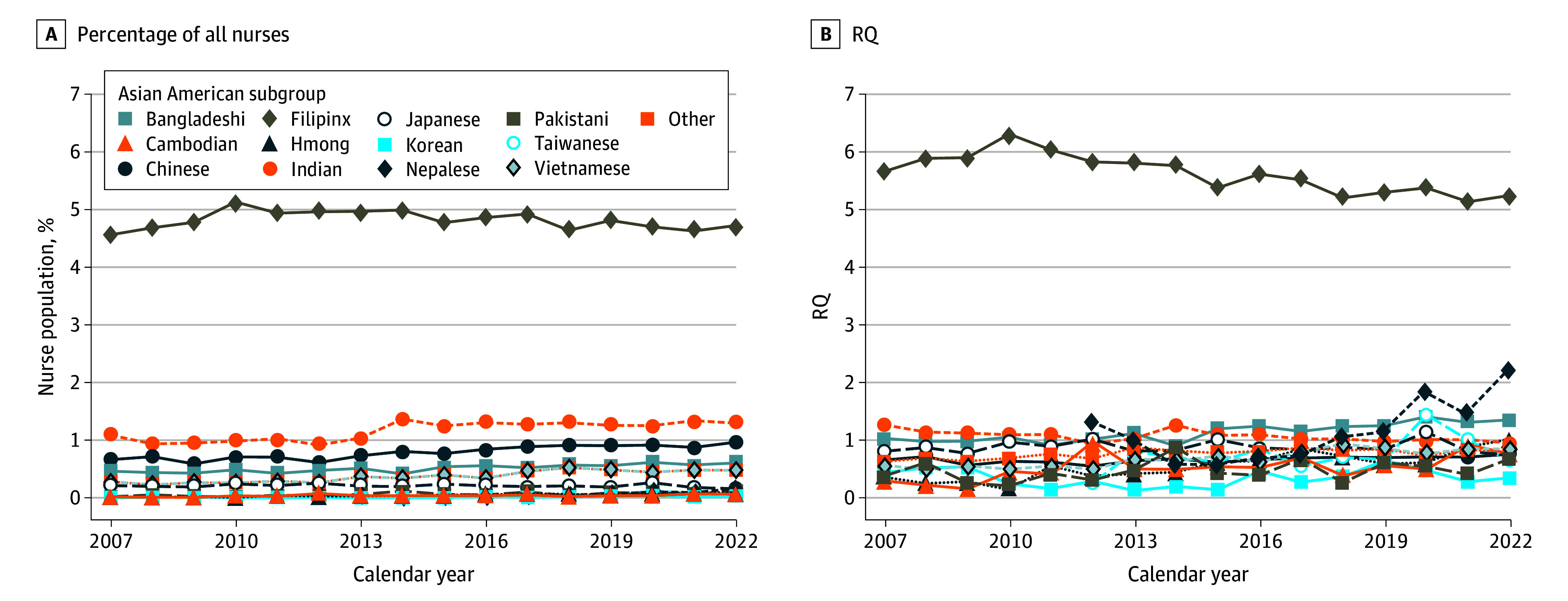
Major Asian American Subgroup Representation Among Registered Nurses, 2007 to 2022 A, Major Asian American subgroups as a percentage of all US registered nurses; B, major Asian American subgroup representation among registered nurses, relative to their representation in the US population. RQ indicates representation quotient.

Among registered nurses, Filipinx Americans made up the largest group across all years; they accounted for 50% of all Asian American RNs (Figure 3A) and had high representation (mean [SD] RQ, 5.63 [0.33]) (Figure 3B). Relative Filipinx representation declined over time (coefficient, −0.6; 95% CI, −0.08 to −0.03); for some groups with an RQ less than 1 (Cambodian, Chinese, Hmong, Other, and Vietnamese), relative representation increased over time but at a small magnitude (coefficients of linear trends <0.05) (eTable 2 in Supplement 1).

**Figure 4.**
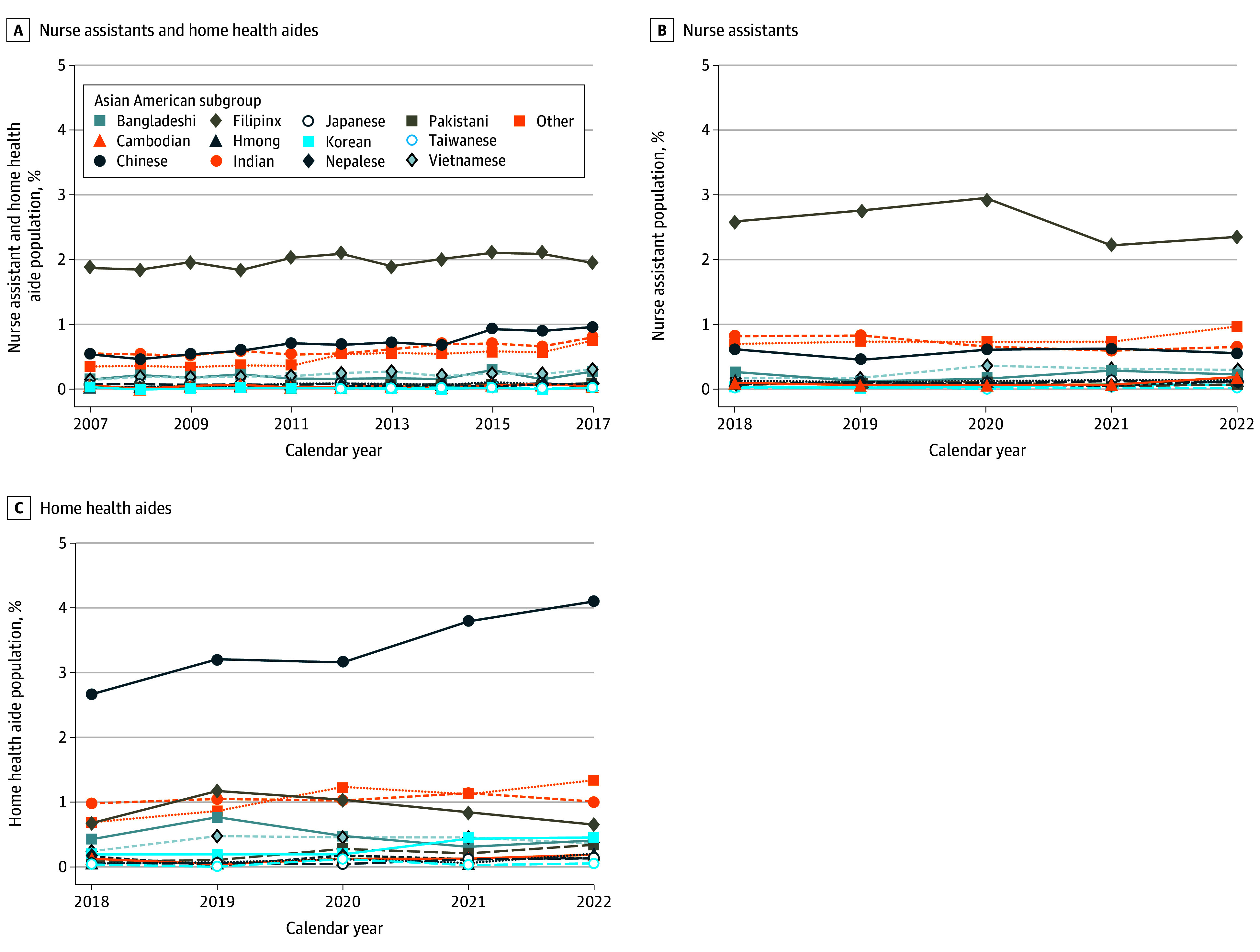
Major Asian American Subgroup Representation Among Registered Nursing Assistants and Home Health Aides, 2007 to 2022 A, Major Asian American subgroups as a percentage of all US nursing assistants and home health aides, 2007 to 2017; B, major Asian American subgroup representation as a percentage of all US nursing assistants, 2018 to 2022; C, major Asian American subgroup representation as a percentage of all US home health aides, 2018 to 2022.

**Figure 5.**
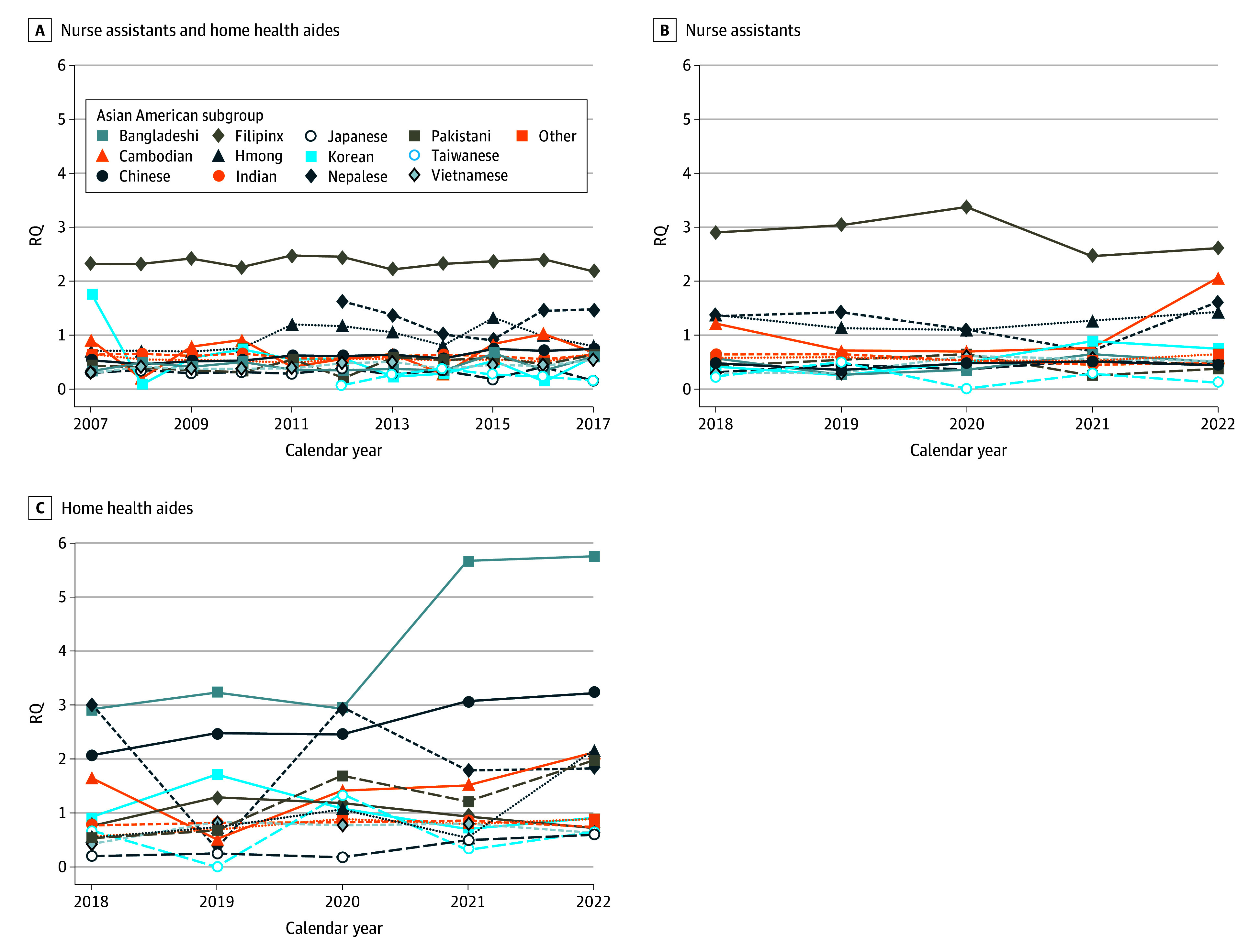
Major Asian American Subgroup Relative Representation Among Registered Nursing Assistants and Home Health Aides, 2007 to 2022 A, Major Asian American subgroup representation among nursing assistants and home health aides combined, relative to their representation in the US population, 2007 to 2017; B, major Asian American subgroup representation among nursing assistants, relative to their representation in the US population, 2018 to 2022; C, major Asian American subgroup representation among home health aides, relative to their representation in the US population, 2018 to 2022. RQ indicates representation quotient.

From 2007 to 2017, major Asian subgroups in the combined category of NAs and HHAs included Filipinx, Chinese, Indian, Vietnamese, and Korean Americans (ie, the major groups in the overall US population) (Figure 4A), with consistently high Filipinx relative representation (mean [SD] RQ, 2.35 [0.10]) (Figure 5A; eTable 3 in Supplement 1). However, when data on the 2 professions were separated in 2018, differences in major subgroups emerged. Among nursing assistants, Filipinx Americans made up the largest group across all years (Figure 4B), at nearly half the Asian American NA workforce, with high relative representation (mean [SD] RQ, 2.87 [0.32]) (Figure 5B; eTable 4 in Supplement 1). Among home health aides, Bangladeshi and Chinese Americans comprised the major share of HHAs (Figure 4C), at nearly half of all Asian American HHAs and high proportional representation (Bangladeshi: mean [SD] RQ, 4.11 [1.48]; Chinese: mean [SD] RQ, 2.66 [0.48]) (Figure 5C; eTable 5 in Supplement 1). Both Bangladeshi and Chinese American relative representation increased from 2018 to 2022 (Bangladeshi: coefficient, 0.81; 95% CI, 0.33-1.29; Chinese: coefficient, 0.29; 95% CI, 0.22-0.36).

## Discussion

Our findings provide an initial view into the diversity of Asian American people both within and between health care occupations. Indian and Pakistani, and Chinese and Taiwanese Americans have had relatively high representation in medicine, whereas Cambodian and Hmong groups representation has remained persistently low. Filipinx Americans have high representation in nursing professions. More recently, Chinese and Bangladeshi Americans have growing representation in home health aides. We discuss potential explanations for differential representation in each occupation, while acknowledging that a more expansive discussion of Asian American history is beyond the scope of this investigation.

Within medicine, Indian and Pakistani Americans comprise over half of all Asian American physicians and have high relative representation in the profession. Our findings are consistent with prior research, which found that Indian-educated immigrants have comprised the largest group of international medical graduate physicians over a similar period of time.^13^ This representation may arise from several factors: first, the Immigration and Nationality Act of 1965 opened immigration pathways in health care and sciences to support both health professions shortages and US Cold War ambitions of technological advancement.^4^ Immigration policies have since evolved to the present landscape of family reunification and expanded health, science, and technology pathways, including the J1, H1B, F-1, and Conrad-30 programs. Furthermore, as a long-run legacy of British imperialism, English is an official language of both India and Pakistan, which may partly explain why these 2 Asian American groups have both high levels of English-language proficiency and low linguistic isolation.^14^ Workers, students and trainees from India have comprised the largest group of highly educated immigrants to the US.^15^ Rather than a model minority narrative, Indian American representation in medicine may reflect a process of hyperselection for highly educated immigrants and their descendants.^16^ Research on access to medical education in India suggests that caste-based structural advantages may further complicate this explanation,^17^ reinforcing the need for greater exploration of the diversity within Asian American subgroups as well.

By contrast, southeast Asian groups are largely underrepresented in medicine, particularly those whose immigration histories reflect adverse selection due to American imperialism. The US prosecution of war, officially in Vietnam and unofficially in Laos and Cambodia, and the postwar conditions that fueled the Cambodian genocide led to large-scale trauma and forced displacement.^4^ The primary pathways for Southeast Asian immigration have been via refugee and asylee programs, rather than those for highly educated immigrants—a hyperselection for those with the greatest vulnerabilities to persecution and socioeconomic disadvantages. Fewer than one-quarter of Hmong and Cambodian Americans have bachelor’s degrees,^18,19^ and those who enter higher education grapple with the myriad challenges of first-generation college students.^20^ For Hmong and Cambodian Americans, their representation among physicians is one-sixth that of their representation in the US population.

Our findings on nursing professions are consistent with the history of US colonization of the Philippines, in which American organizations built hospitals and nursing schools in the 20th century to “civilize” the Filipinx population. Since then, the US has repeatedly turned to English-speaking, Western systems-educated nurses in the Philippines to fill domestic nursing shortages, from World War II to the encouragement of labor exportation under the Marcos dictatorship through the 1980s.^21^ The high representation of Filipinx Americans in nursing professions reflects systematic professional selection with particularly detrimental consequences. Filipinx nurses disproportionately work in underserved communities, in systems with fewer resources, and in high-intensity services^22^; in the initial years of the COVID-19 pandemic, Filipinx health care workers had substantially higher case and mortality rates than White and Asian American health care workers—both in health care and in the general population.^23^

Lastly, Asian American representation in the NA and HHA professions further emphasize the need to understand the socioeconomic diversity within and between Asian American subgroups. Both Bangladeshi and Chinese Americans have become overrepresented among HHAs. The differential representation of Bangladeshi Americans in a low-wage health support occupation reinforces the need to examine Bangladeshi Americans as distinct from other South Asian groups. Major considerations include the roles of the 1970s American-supported genocide of Bangladeshis, carried out by then-West Pakistan,^24^ and the post-1990 US immigration Diversity Program (based on previous low migration, not technical skills or family reunification).^25^ Our findings also point to the need for greater examination of the contribution of health care stratification to vast in-group economic inequality among Chinese Americans, given their predominance in both high and low status health professions.^26^

Our findings complicate the dialogue on health care workforce diversity and representation. The narrative of Asian American overrepresentation in the health professions applies to only a few subgroups; the mismatch between narrative and data can be traced in part to racialization of who is Asian American.^27^ In 1968, activists Emma Gee and Yuji Ichioka promoted the term *Asian American* to advocate for cohesive political power and strengthen multiracial solidarity in support of the civil rights movement.^4^ In the decades since, health care institutions have repurposed Asian American to denote a racialized group, not a political coalition.

The practice of lumping over 40 ethnoracialized groups under 1 label reflects the process of Asianization, the assumption of a homogenous race that ignores Asian American diversity and wipes clean the more troubling histories of subgroups who exist due to American colonization and war, displacement, and trauma.^28^ Asianization also denies the structural racism of immigration policy that advantages some, showing up as high physician representation, and contributes to labor and economic exploitation of others in the direct care workforce.^29^

In 2023, in the case of *Students for Fair Admissions vs Harvard*, the US Supreme Court struck down race considerations in higher education admissions, claiming (without evidence) that such practices discriminated against Asian American individuals.^30^ The ruling undermined efforts toward advancing health workforce diversity and diverted attention from the substantiated issues: in admissions, Asian American populations are harmed by negative action, such as practices that favor White applicants, and that Asianization and the model minority stereotype hide the challenges of more disadvantaged subgroups.^31^ The plaintiffs deliberately excluded Southeast Asian American individuals from their definition of Asian American, demonstrating how the umbrella term perpetuates erasure.^32^ As administrators and educators look to revise diversity, equity and inclusion programs to fit new legal and judicial requirements, they would benefit from more data, not less.

### Implications

Applicant and workforce data disaggregation constitute an important first step in antiracist practice.^5^ Examination by both occupation and disaggregated subgroups counters blanket assumptions of exceptional Asian American educational attainment and income. We join the calls upon institutions to collect and report disaggregated workforce data, following at minimum the Office of Management and Budget guidance.^11^ Whereas the lack of subgroup information continues to stymie documentation of Asian American health disparities,^6^ some professional organizations, including the Association of American Medical Colleges,^33^ systematically collect subgroup data already. Furthermore, current discussion and practices fail to account for the largest and fastest growing Asian American group, those with multiple ethnoracial identities, whose health workforce representation is roughly proportional or slightly below their population representation.

Second, health care organizations need to strengthen holistic evaluation approaches^34,35,36^ to account for differential selection of Asian American populations by historical and policy processes that occur outside of academic institutions. By recognizing these differences, leaders are better positioned to recognize the assets brought by Asian American health care professionals, including linguistic and cultural expertise, experiences that can inform care for complex needs of trauma, poverty, and displacement, and commitment to these communities.

Third, the health care professions must attend to health care justice. The patterns of ethnic-specific overrepresentation and underrepresentation within socioeconomically stratified health care occupations are no accident, but rather reflect historical forces of imperialism and neoliberalism. For the most impacted Asian American communities, their respective inequities are rooted in state-sanctioned, violent actions, and continue to be shaped by contemporary geopolitical forces. Engaging in health justice means acknowledging and naming the Western powers, including the US, who fostered the migration patterns that have produced current Asian American inequities. Health care justice also includes the critical need to raise wages for those working in health support occupations. In 2019, median earnings for women employed in health occupations was lowest for nursing assistants, at $28 686; for men, under $30 000 for HHAs.^37^ When groups such as Bangladeshi and Chinese Americans are overrepresented in these occupations, health care workforce inequities may thus drive the wide income inequities among Asian American groups.^38^

The current language of diversity and representation in the health care workforce adopts a mythology of neutrality and ahistoricism. Contemporary Asian American diversity reflects multiple distinct histories of Western and Asian colonialism and imperialism. The major inequities between and within Asian subgroups are not merely happenstance descriptive conditions, but rooted in policies and practices that produce the current demographic landscape. Future discussions require a critical race lens that interrogates and disrupts current narratives that hinder meaningful discussion about Asian American populations and workforce diversity. Updated narratives will help to explain how higher-earning professions, such as medicine, continue to systematically exclude some Asian subgroups.

### Limitations

This study has limitations. First, because we analyzed ACS data, we are unable to examine more detailed information on individuals, such as international vs US training and patient care vs teaching; however, ACS allows us to capture multiple professions and subgroups nationally over time. ACS also includes hours worked and setting, which could be considered in conjunction with other professional data sources in future studies. Second, as noted, we were unable to examine other groups such as Afghan Americans, whose experiences of war and displacement, health, and workforce needs also merit further investigation. Third, multiracial and multiethnic Asian American individuals are the fastest growing subgroups in the U.S, but much-needed critical work remains on how they can be characterized in the health workforce.^39^ Fourth, we present broad categories with the understanding that greater attention is needed to intersectional identities, such as gender and caste. Lastly, we present a limited set of professions for the purposes of emphasizing the complexity between and within occupational categories; ongoing disaggregated data analysis is needed for the multitude of other health professions, from dentistry and pharmacy to laboratory and radiology technicians.

## Conclusions

To understand the landscape of workforce diversity and how to appropriately characterize Asian American populations, health care leaders need to acknowledge Asian American diversity, including severe intragroup inequities, by systematic data disaggregation and reporting. Our findings highlight the need to conduct ongoing investigation and reflection on how historical and contemporary structural racism shape workforce representation for Asian American individuals and other ethnic groups. Recent political opposition to diversity, equity, and inclusion does not change the reality that the current health care workforce neither adequately serves nor represents the American populations in greatest need. Implementing sustainable solutions to transform the workforce will require clear recognition of underlying sources of oppression and political power imbalances.

## Notes

Supplement 1. **eTable 1.** Asian American Subgroups: Percentage of All US Physicians, of All Asian American Physicians, Representation Quotient (RQ) and Linear Trends, 2007 to 2022 **eTable 2. **Asian American Subgroups: Percentage of All US Registered Nurses, of All Asian American Registered Nurses, Representation Quotient and Linear Trends, 2007 to 2022 **eTable 3. **Asian American Subgroups: Percentage of All US Nursing Assistants and Home Health Aides, of All Asian American Nursing Assistants and Home Health Aides, Representation Quotient and Linear Trends, 2007 to 2017 **eTable 4. **Asian American Subgroups: Percentage of All US Nursing Assistants, of All Asian American Nursing Assistants, Representation Quotient and Linear Trends, 2018 to 2022 **eTable 5.** Asian American Subgroups: Percentage of All US Home Health Aides, of All Asian American Home Health Aides, Representation Quotient and Linear Trends, 2018 to 2022 **eAppendix. **Items Used From the American Community Survey for Analyses

Supplement 2.
Data Sharing Statement
