# Supplementary material for: Asian American Diversity and Representation in the Health Care Workforce, 2007 to 2022
Source: JAMA Netw Open. 2024 Oct 17;7(10):e2440071. doi: 10.1001/jamanetworkopen.2024.40071 (PMC11581573; doi:10.1001/jamanetworkopen.2024.40071)
Supplement: Supplement 1. — eTable 1. Asian American Subgroups: Percentage of All US Physicians, of All Asian American Physicians, Representation Quotient (RQ) and Linear Trends, 2007 to 2022 eTable 2. Asian American Subgroups: Percentage of All US Registered Nurses, of All Asian American Registered Nurses, Representation Quotient and Linear Trends, 2007 to 2022 eTable 3. Asian American Subgroups: Percentage of All US Nursing Assistants and Home Health Aides, of All Asian American Nursing Assistants and Home Health Aides, Representation Quotient and Linear Trends, 2007 to 2017 eTable 4. Asian American Subgroups: Percentage of All US Nursing Assistants, of All Asian American Nursing Assistants, Representation Quotient and Linear Trends, 2018 to 2022 eTable 5. Asian American Subgroups: Percentage of All US Home Health Aides, of All Asian American Home Health Aides, Representation Quotient and Linear Trends, 2018 to 2022 eAppendix. Items Used From the American Community Survey for Analyses [file jamanetwopen-e2440071-s001.pdf]

Supplemental Online Content

Ko M, Dinh K, Iv S, Hahn M. Asian American diversity and representation in the health care workforce, 2007 to 2022. *JAMA Netw Open*. 2024;7(10):e2440071. doi:10.1001/jamanetworkopen.2024.40071

eTable 1. Asian American Subgroups: Percentage of All US Physicians, of All Asian American Physicians, Representation Quotient (RQ) and Linear Trends, 2007 to 2022
eTable 2. Asian American Subgroups: Percentage of All US Registered Nurses, of All Asian American Registered Nurses, Representation Quotient and Linear Trends, 2007 to 2022
eTable 3. Asian American Subgroups: Percentage of All US Nursing Assistants and Home Health Aides, of All Asian American Nursing Assistants and Home Health Aides, Representation Quotient and Linear Trends, 2007 to 2017
eTable 4. Asian American Subgroups: Percentage of All US Nursing Assistants, of All Asian American Nursing Assistants, Representation Quotient and Linear Trends, 2018 to 2022
eTable 5. Asian American Subgroups: Percentage of All US Home Health Aides, of All Asian American Home Health Aides, Representation Quotient and Linear Trends, 2018 to 2022
eAppendix. Items Used From the American Community Survey for Analyses

This supplemental material has been provided by the authors to give readers additional information about their work.

© 2024 Ko M et al. *JAMA Network Open*.

eTable 1. Asian American Subgroups: Percentage of All US Physicians, of All Asian American Physicians, Representation Quotient (RQ) and Linear Trends, 2007 to 2022. (Asian American physicians: mean N=206,844 per year**)**

	% of US physicians		% of AA physicians		RQ		RQ Linear Trend (years)		
**Subgroup**	**Mean**	**SD**	**Mean**	**SD**	**Mean**	**SD**	**Coefficient**	**95% CI**	
**Bangladeshi**	0.125	0.047	0.599	0.222	2.552	0.956	-0.102	-0.183	-0.021
**Bhutanese**	0.000	0.002	0.003	0.010	0.074	0.245	0.022	-0.031	0.075
**Cambodian**	0.018	0.015	0.087	0.074	0.227	0.195	0.001	-0.026	0.028
**Chinese**	3.920	0.286	18.922	1.389	3.330	0.228	-0.032	-0.050	-0.013
**Filipinx**	1.772	0.236	8.629	1.657	2.075	0.340	-0.060	-0.080	-0.040
**Hmong**	0.017	0.018	0.079	0.091	0.195	0.228	-0.002	-0.026	0.022
**Indian**	8.435	0.736	40.599	1.613	7.846	0.917	-0.174	-0.222	-0.126
**Indonesian**	0.034	0.017	0.166	0.093	1.518	0.805	-0.069	-0.174	0.035
**Japanese**	0.492	0.096	2.377	0.471	2.037	0.374	0.013	-0.034	0.061
**Korean**	1.454	0.121	7.034	0.742	3.207	0.277	0.006	-0.023	0.034
**Laotian**	0.015	0.014	0.071	0.067	0.252	0.242	0.008	-0.018	0.034
**Malaysian**	0.016	0.015	0.078	0.076	2.900	3.320	-0.078	-0.524	0.367
**Mongolian**	0.003	0.004	0.017	0.022	0.664	0.942	-0.091	-0.283	0.101
**Myanma**	0.058	0.049	0.387	0.153	1.601	0.512	0.074	-0.029	0.177
**Nepalese**	0.095	0.084	0.635	0.290	2.939	1.560	-0.204	-0.599	0.192
**Other**	1.679	0.475	7.957	1.730	1.657	0.205	-0.054	-0.157	0.048
**Pakistani**	1.181	0.273	5.639	0.994	8.930	0.957	-0.043	-0.315	0.229
**Sri Lankan**	0.111	0.045	0.533	0.210	7.776	2.880	-0.120	-0.312	0.073
**Taiwanese**	0.318	0.237	2.122	0.451	7.886	1.439	-0.055	-0.139	0.029
**Thai**	0.163	0.057	0.791	0.290	2.807	0.971	0.009	-0.017	0.036
**Vietnamese**	0.887	0.151	4.264	0.638	1.638	0.245	-0.012	-0.034	0.010

*The following groups were not separately identified by the Census Bureau until 2012-2022: Bhutanese, Mongolian, Myanma, Nepalese, Taiwanese

© 2024 Ko M et al. *JAMA Network Open*.

eTable 2. Asian American Subgroups: Percentage of All US Registered Nurses, of All Asian American Registered Nurses, Representation Quotient and Linear Trends, 2007 to 2022.

	% of US registered nurses		% of AA registered nurses		RQ		RQ Linear Trend (years)		
**Subgroup**	**Mean**	**SD**	**Mean**	**SD**	**Mean**	**SD**	**Coefficient**	**95% CI**	
**Bangladeshi**	0.018	0.010	0.189	0.093	0.349	0.157	0.001	-0.016	0.018
**Bhutanese**	0.003	0.005	0.045	0.059	0.567	0.730	0.064	-0.055	0.183
**Cambodian**	0.043	0.019	0.462	0.200	0.529	0.226	0.028	0.010	0.047
**Chinese**	0.792	0.117	8.658	0.655	0.668	0.056	0.007	0.001	0.013
**Filipinx**	4.827	0.159	53.372	5.232	5.634	0.335	-0.058	-0.084	-0.031
**Hmong**	0.049	0.026	0.524	0.249	0.554	0.244	0.044	0.029	0.060
**Indian**	1.167	0.163	12.767	1.022	1.076	0.096	-0.013	-0.019	-0.007
**Indonesian**	0.031	0.011	0.345	0.117	1.399	0.464	0.001	-0.059	0.060
**Japanese**	0.214	0.028	2.370	0.402	0.886	0.105	0.001	-0.014	0.015
**Korean**	0.516	0.066	5.657	0.425	1.140	0.158	0.028	0.020	0.036
**Laotian**	0.036	0.012	0.398	0.135	0.594	0.184	0.010	-0.012	0.031
**Malaysian**	0.005	0.005	0.054	0.053	0.827	0.790	0.026	-0.069	0.121
**Mongolian**	0.004	0.006	0.066	0.064	0.980	0.969	0.153	-0.023	0.329
**Myanma**	0.009	0.008	0.130	0.062	0.235	0.098	0.017	-0.007	0.041
**Nepalese**	0.042	0.047	0.631	0.428	1.155	0.530	0.104	-0.008	0.216
**Other**	0.065	0.281	8.623	2.408	0.772	0.081	0.013	-0.005	0.030
**Pakistani**	0.008	0.032	0.700	0.311	0.476	0.183	0.031	-0.008	0.070
**Sri Lankan**	0.033	0.007	0.089	0.071	0.583	0.474	0.044	-0.022	0.111
**Taiwanese**	0.074	0.034	0.490	0.306	0.753	0.296	0.027	-0.014	0.067
**Thai**	0.374	0.026	0.807	0.243	1.253	0.354	0.027	0.020	0.035
**Vietnamese**	0.804	0.099	4.049	0.773	0.685	0.150	0.012	0.005	0.018

*The following groups were not separately identified by the Census Bureau until 2012-2022: Bhutanese, Mongolian, Myanma, Nepalese, Taiwanese

© 2024 Ko M et al. *JAMA Network Open*.

eTable 3. Asian American Subgroups: Percentage of All US Nursing Assistants and Home Health Aides, of All Asian American Nursing Assistants and Home Health Aides, Representation Quotient and Linear Trends, 2007 to 2017.

	% of US NAs & HHAs		% of AA NAs & HHAs		RQ		RQ Linear Trend (years)		
**Subgroup**	**Mean**	**SD**	**Mean**	**SD**	**Mean**	**SD**	**Coefficient**	**95% CI**	
**Bangladeshi**	0.022	0.012	0.466	0.262	0.559	0.454	-0.063	-0.193	0.067
**Bhutanese**	0.005	0.006	0.178	0.123	1.095	0.605	0.107	-0.400	0.614
**Cambodian**	0.053	0.022	1.122	0.481	0.665	0.272	0.011	-0.054	0.077
**Chinese**	0.707	0.167	14.862	1.718	0.614	0.096	0.026	0.016	0.035
**Filipinx**	1.975	0.099	42.454	4.226	2.347	0.096	-0.006	-0.026	0.014
**Hmong**	0.075	0.024	1.575	0.381	0.934	0.227	0.044	0.029	0.060
**Indian**	0.621	0.090	13.216	0.948	0.622	0.037	-0.004	-0.011	0.004
**Indonesian**	0.032	0.017	0.686	0.352	1.501	0.752	0.012	-0.151	0.175
**Japanese**	0.076	0.019	0.017	0.005	0.308	0.076	-0.007	-0.027	0.013
**Korean**	0.202	0.051	4.303	0.955	0.442	0.114	0.011	-0.014	0.037
**Laotian**	0.042	0.014	0.903	0.307	0.669	0.232	0.007	-0.033	0.047
**Malaysian**	0.005	0.005	0.101	0.107	0.888	0.920	0.098	-0.028	0.224
**Mongolian**	0.005	0.010	0.222	0.228	1.711	1.983	-0.351	-1.006	0.304
**Myanma**	0.017	0.020	0.498	0.220	0.654	0.288	0.102	-0.003	0.208
**Nepalese**	0.029	0.030	0.917	0.202	1.313	0.285	-0.019	-0.202	0.164
**Other**	0.491	0.136	10.282	1.668	0.564	0.053	0.022	0.005	0.040
**Pakistani**	0.058	0.026	1.201	0.429	0.468	0.135	0.069	-0.026	0.163
**Sri Lankan**	0.014	0.007	0.289	0.127	1.065	0.501	0.008	-0.075	0.090
**Taiwanese**	0.006	0.007	0.239	0.111	0.233	0.105	-0.009	-0.059	0.041
**Thai**	0.041	0.016	0.889	0.367	0.728	0.276	0.015	0.004	0.026
**Vietnamese**	0.227	0.047	4.797	0.584	0.428	0.071	0.000	-0.017	0.017

*The following groups were not separately identified by the Census Bureau until 2012-2022: Bhutanese, Mongolian, Myanma, Nepalese, Taiwanese

© 2024 Ko M et al. *JAMA Network Open*.

eTable 4. Asian American Subgroups: Percentage of All US Nursing Assistants, of All Asian American Nursing Assistants, Representation Quotient and Linear Trends, 2018 to 2022.

	% of US NAs		% of AA NAs		RQ		RQ Linear Trend (years)		
**Subgroup**	**Mean**	**SD**	**Mean**	**SD**	**Mean**	**SD**	**Coefficient**	**95% CI**	
**Bangladeshi**	0.040	0.022	0.696	0.408	0.553	0.255	0.130	-0.026	0.285
**Bhutanese**	0.009	0.006	0.149	0.112	1.163	0.820	-0.197	-0.988	0.593
**Cambodian**	0.089	0.052	1.548	0.884	1.082	0.582	0.173	-0.506	0.852
**Chinese**	0.566	0.071	9.831	1.376	0.444	0.060	0.008	-0.052	0.067
**Filipinx**	2.569	0.297	44.508	3.915	2.874	0.360	-0.116	-0.328	0.097
**Hmong**	0.123	0.012	2.136	0.255	1.252	0.146	0.025	-0.149	0.198
**Indian**	0.704	0.106	12.213	1.843	0.544	0.091	-0.052	-0.092	-0.012
**Indonesian**	0.036	0.009	0.630	0.172	1.453	0.325	-0.092	-0.421	0.238
**Japanese**	0.094	0.017	1.648	0.363	0.414	0.086	0.037	-0.025	0.098
**Korean**	0.206	0.070	3.595	1.328	0.459	0.156	0.025	-0.133	0.182
**Laotian**	0.030	0.018	0.519	0.307	0.552	0.318	-0.113	-0.398	0.172
**Malaysian**	0.002	0.005	0.035	0.078	0.257	0.574	0.257	-0.260	0.773
**Mongolian**	0.013	0.015	0.222	0.252	1.948	2.084	-0.030	-2.824	2.763
**Myanma**	0.045	0.018	0.784	0.355	0.748	0.278	-0.013	-0.349	0.324
**Nepalese**	0.076	0.027	1.312	0.445	1.231	0.350	-0.020	-0.429	0.388
**Other**	0.767	0.024	13.306	1.841	0.566	0.050	-0.039	-0.148	0.069
**Pakistani**	0.071	0.005	1.216	0.369	0.440	0.153	-0.035	-0.482	0.412
**Sri Lankan**	0.006	0.011	0.105	0.092	0.376	0.372	-0.045	-0.169	0.080
**Taiwanese**	0.014	0.012	0.253	0.203	0.220	0.186	0.090	-0.106	0.286
**Thai**	0.047	0.088	0.822	0.199	0.781	0.231	0.071	-0.006	0.147
**Vietnamese**	0.258	0.111	4.472	1.498	0.451	0.149	0.007	-0.050	0.064

© 2024 Ko M et al. *JAMA Network Open*.

eTable 5. Asian American Subgroups: Percentage of All US Home Health Aides, of All Asian American Home Health Aides, Representation Quotient and Linear Trends, 2018 to 2022.

	% of US HHAs		% of AA HHAs		RQ		RQ Linear Trend (years)		
**Subgroup**	**Mean**	**SD**	**Mean**	**SD**	**Mean**	**SD**	**Coefficient**	**95% CI**	
**Bangladeshi**	0.292	0.138	3.392	1.270	4.106	1.478	0.812	0.332	1.291
**Bhutanese**	0.041	0.055	0.485	0.661	5.433	7.072	0.812	0.332	1.291
**Cambodian**	0.119	0.052	1.419	0.581	1.441	0.585	0.193	-0.433	0.818
**Chinese**	3.384	0.566	40.123	2.424	2.659	0.477	0.290	0.219	0.360
**Filipinx**	0.874	0.226	10.504	2.780	0.977	0.253	-0.042	-0.351	0.266
**Hmong**	0.098	0.063	1.128	0.600	1.007	0.677	0.304	-0.254	0.862
**Indian**	1.039	0.061	12.506	1.656	0.802	0.047	-0.001	-0.063	0.061
**Indonesian**	0.023	0.009	0.271	0.096	0.925	0.349	0.101	-0.158	0.360
**Japanese**	0.077	0.041	0.894	0.378	0.342	0.190	0.104	0.038	0.170
**Korean**	0.476	0.173	5.773	2.258	1.065	0.387	-0.104	-0.487	0.278
**Laotian**	0.043	0.042	0.571	0.650	0.738	0.685	-0.311	-0.805	0.183
**Malaysian**	0.001	0.003	0.014	0.031	0.192	0.429	0.096	-0.325	0.517
**Mongolian**	0.011	0.014	0.126	0.166	1.771	2.359	-0.337	-2.675	2.002
**Myanma**	0.089	0.072	1.025	0.705	1.443	1.091	0.510	-0.467	1.488
**Nepalese**	0.121	0.060	1.473	0.828	1.990	1.101	-0.096	-1.100	0.908
**Other**	1.048	0.111	12.299	1.841	0.765	0.138	0.342	0.167	0.516
**Pakistani**	0.201	0.022	2.295	1.065	1.216	0.625	0.462	-0.227	1.151
**Sri Lankan**	0.014	0.045	0.150	0.243	0.701	1.062	0.025	-0.263	0.313
**Taiwanese**	0.046	0.026	0.545	0.518	0.598	0.505	0.145	-0.240	0.529
**Thai**	0.029	0.099	0.330	0.269	0.478	0.441	0.039	-0.171	0.249
**Vietnamese**	0.396	0.269	4.677	0.948	0.692	0.169	0.075	0.024	0.126

© 2024 Ko M et al. *JAMA Network Open*.

eAppendix. Items Used From the American Community Survey for Analyses.

The American Community Survey
On ascertaining race: “The data on race are based on self-identification and the categories on the form generally reflect a social definition of race. The categories are not an attempt to define race biologically, anthropologically, or genetically. Respondents can mark more than one race on the form to indicate their racial mixture.”

Survey Questions:
2007 - Race:
6 Form. What is this person's race? Mark (X) one or more races to indicate what this person considers himself/herself to be
[ ] White
[ ] Black, African Am., or Negro
[ ] American Indian or Alaska Native -- Print name of enrolled or principal tribe -->
____
[ ] Asian Indian
[ ] Chinese
[ ] Filipino
[ ] Japanese
[ ] Korean
[ ] Vietnamese
[ ] Other Asian - Print race --> ____________________
[ ] Native Hawaiian
[ ] Guamanian or Chamorro
[ ] Samoan
[ ] Other Pacific Islander
[ ] Some other race - Print race below --> ____________________

6 Instructions. Mark one or more categories to indicate what each person considers himself or herself to be.
If you mark the "American Indian or Alaska Native" box, also print the name of the tribe(s) in which the person is enrolled. If the person is not enrolled in a tribe, print the name of the principal tribe.
If you mark the "Other Asian" or the "Other Pacific Islander" box, print the name of the specific race(s) or group(s) in the space provided.
The category Other Asian includes persons who identify themselves as Burmese, Hmong, Indonesian, Laotian, Pakistani, Thai, Cambodian, Sri Lankan, and so on.
The category Other Pacific Islander includes persons who identify themselves as Fijian, Tongan, Polynesian, Tahitian, and so on.
If you mark the "Some other race" box, print the race(s) or group(s) in the space provided.
This question should be answered for ALL persons, regardless of citizenship status.

2008 - Race:

© 2024 Ko M et al. *JAMA Network Open*.

6. What is Person X's race? Mark (X) one or more boxes.
[ ] White
[ ] Black, African Am., or Negro
[ ] American Indian or Alaska Native -- Print name of enrolled or principal tribe. -->
____
[ ] Asian Indian
[ ] Japanese
[ ] Chinese
[ ] Korean
[ ] Filipino
[ ] Vietnamese
[ ] Other Asian -- Print race, for example, Hmong, Laotian Thai, Pakistani, Cambodian and so on_____________________________________
[ ] Native Hawaiian
[ ] Guamanian or Chamorro
[ ] Samoan
[ ] Other Pacific Islander ? Print race, for example,Fijian, Tongan, and so on. --
____
[ ] Some other race -- Print race. -->
____

6. Mark one or more races.
If you mark the "American Indian or Alaska Native" box, also print the name of the tribe(s) in which the person is enrolled. If the person is not enrolled in a tribe, print the name of the principal tribe.
If you mark the "Other Asian" or the "Other Pacific Islander" box, print the name of the specific race(s) or group(s) in the space provided.
The category "Other Asian" includes persons who identify themselves as Laotian, Thai, Pakistani, Cambodian, and so on.
The category "Other Pacific Islander" includes persons who identify themselves as Fijian, Tongan, and so on.
If you mark the "Some other race" box, print the race(s) or group(s) in the space provided. This question should be answered by ALL persons.

2009 - Race:
6. What is Person 1's race? Mark (X) one or more boxes.
[ ] White
[ ] Black, African Am., or Negro
[ ] American Indian or Alaska Native -- Print name of enrolled or principal tribe.
____
[ ] Asian Indian
[ ] Japanese
[ ] Chinese
[ ] Korean
[ ] Filipino
[ ] Vietnamese

© 2024 Ko M et al. *JAMA Network Open.*

[ ] Other Asian -- Print race, for example, Hmong, Laotian, Thai, Pakistani, Cambodian, and so on. ___________________________________
[ ] Native Hawaiian
[ ] Guamanian or Chamorro
[ ] Samoan
[ ] Other Pacific IslanderPrint race, for example,Fijian, Tongan, and so on.
____
[ ] Some other race -- Print race.
____

6. Mark all boxes for the appropriate races.
The concept of race, as used by the Census Bureau, reflects self-identification by individuals according to the race or races with which they identify.
The instruction before question 5, "For this survey, Hispanic origins are not races" reflects the federal government's treatment of Hispanic origin and race as separate and distinct concepts. People who identify their origin as Hispanic, Latino, or Spanish may be of any race.
People may choose to provide two or more races either by marking two or more race response boxes, by providing multiple write-in responses, or by some combination of marking boxes and writing in responses.
If you mark the "American Indian or Alaska Native" box, also print the name of the tribe(s) in which the person is enrolled. If the person is not enrolled in a tribe, print the name of the principal tribe.
If you mark the "Other Asian" box, print the name of the specific race(s) or group(s) in the space provided. The category "Other Asian" includes persons who identify themselves as Laotian, Thai, Pakistani, Cambodian, Sri Lankan, and so on.
If you mark the "Other Pacific Islander" box, print the name of the specific race(s) or group(s) in the space provided. The category "Other Pacific Islander" includes persons who identify themselves as Fijian, Tongan, Polynesian, Tahitian and so on.
If you mark the "Some other race" box, print the race(s) or group(s) in the space provided.
This question should be answered by ALL persons.

2010 - Race:
6. What is Person 1's race? Mark (X) one or more boxes.
[ ] White
[ ] Black, African Am., or Negro
[ ] American Indian or Alaska Native -- Print name of enrolled or principal tribe. ?
____
[ ] Asian Indian
[ ] Japanese
[ ] Chinese
[ ] Korean
[ ] Filipino
[ ] Vietnamese
[ ] Other Asian -- Print race .for example, Hmong, Laotian, Thai, Pakistani, Cambodian and so on. ? ____________________
[ ] Native Hawaiian
[ ] Guamanian or Chamorro

© 2024 Ko M et al. *JAMA Network Open*.

[ ] Samoan
[ ] Other Pacific Islander ? Print race, for example, Fijian, Tongan, and so on. ?
____
[ ] Some other race -- Print race. ?
____

6.Mark all boxes for the appropriate races.
The concept of race, as used by the Census Bureau, reflects self-identification by individuals according to the race or races with which they identify.
The instruction before question 5, "For this survey, Hispanic origins are not races" reflects the federal government's treatment of Hispanic origin and race as separate and distinct concepts. People who identify their origin as Hispanic, Latino, or Spanish may be of any race.
People may choose to provide two or more races either by marking two or more race response boxes, by providing multiple write-in responses, or by some combination of marking boxes and writing in responses.
If you mark the "American Indian or Alaska Native" box, also print the name of the tribe(s) in which the person is enrolled. If the person is not enrolled in a tribe, print the name of the principal tribe.
If you mark the "Other Asian" box, print the name of the specific race(s) or group(s) in the space provided. The category "Other Asian" includes persons who identify themselves as Laotian, Thai, Pakistani, Cambodian, Sri Lankan, and so on.
If you mark the "Other Pacific Islander" box, print the name of the specific race(s) or group(s) in the space provided. The category "Other Pacific Islander" includes persons who identify themselves as Fijian, Tongan, Polynesian, Tahitian and so on.
If you mark the "Some other race" box, print the race(s) or group(s) in the space provided.
This question should be answered by ALL persons.

2011 - Race:
6. What is Person X's race? Mark (X) one or more boxes.
[ ] White
[ ] Black, African Am., or Negro
[ ] American Indian or Alaska Native -- Print name of enrolled or principal tribe. -->
____
[ ] Asian Indian
[ ] Japanese
[ ] Chinese
[ ] Korean
[ ] Filipino
[ ] Vietnamese
[ ] Other Asian -- Print race, for example, Hmong, Laotian, Thai, Pakistani, Cambodian and so on.
[ ] Native Hawaiian
[ ] Guamanian or Chamorro
[ ] Samoan
[ ] Other Pacific Islander ?Print race, for example, Fijian, Tongan, and so on.
[ ] Some other race -- Print race. -->

© 2024 Ko M et al. *JAMA Network Open*.

____

6.Mark all boxes for the appropriate races.
The concept of race, as used by the Census Bureau, reflects self-identification by individuals according to the race or races with which they identify.
The instruction before question 5, "For this survey, Hispanic origins are not races" reflects the federal government's treatment of Hispanic origin and race as separate and distinct concepts. People who identify their origin as Hispanic, Latino, or Spanish may be of any race.
People may choose to provide two or more races either by marking two or more race response boxes, by providing multiple write-in responses, or by some combination of marking boxes and writing in responses.
If you mark the "American Indian or Alaska Native" box, also print the name of the tribe(s) in which the person is enrolled. If the person is not enrolled in a tribe, print the name of the principal tribe.
If you mark the "Other Asian" box, print the name of the specific race(s) or group(s) in the space provided. The category "Other Asian" includes persons who identify themselves as Laotian, Thai, Pakistani, Cambodian, Sri Lankan, and so on.
If you mark the "Other Pacific Islander" box, print the name of the specific race(s) or group(s) in the space provided. The category "Other Pacific Islander" includes persons who identify themselves as Fijian, Tongan, Polynesian, Tahitian and so on.
If you mark the "Some other race" box, print the race(s) or group(s) in the space provided.
This question should be answered by ALL persons.

2012 - Race:
6. What is Person 1's race? Mark (X) one or more boxes.
[ ] White
[ ] Black, African Am., or Negro
[ ] American Indian or Alaska Native -- Print name of enrolled or principal tribe.
____
[ ] Asian Indian
[ ] Japanese
[ ] Chinese
[ ] Korean
[ ] Filipino
[ ] Vietnamese
[ ] Other Asian -- Print race, for example, Hmong, Laotian, Thai, Pakistani, Cambodian, and so on. ________________________________
[ ] Native Hawaiian
[ ] Guamanian or Chamorro
[ ] Samoan
[ ] Other Pacific IslanderPrint race, for example,Fijian, Tongan, and so on.
____
[ ] Some other race -- Print race.
____

6.Mark all boxes for the appropriate races.

© 2024 Ko M et al. *JAMA Network Open.*

The concept of race, as used by the Census Bureau, reflects self-identification by individuals according to the race or races with which they identify.
The instruction before question 5, "For this survey, Hispanic origins are not races" reflects the federal government's treatment of Hispanic origin and race as separate and distinct concepts. People who identify their origin as Hispanic, Latino, or Spanish may be of any race.
People may choose to provide two or more races either by marking two or more race response boxes, by providing multiple write-in responses, or by some combination of marking boxes and writing in responses.
If you mark the "American Indian or Alaska Native" box, also print the name of the tribe(s) in which the person is enrolled. If the person is not enrolled in a tribe, print the name of the principal tribe.
If you mark the "Other Asian" box, print the name of the specific race(s) or group(s) in the space provided. The category "Other Asian" includes persons who identify themselves as Laotian, Thai, Pakistani, Cambodian, Sri Lankan, and so on.
If you mark the "Other Pacific Islander" box, print the name of the specific race(s) or group(s) in the space provided. The category "Other Pacific Islander" includes persons who identify themselves as Fijian, Tongan, Polynesian, Tahitian and so on.
If you mark the "Some other race" box, print the race(s) or group(s) in the space provided.
This question should be answered by ALL persons.

2013 - Race:
6. What is Person X's race? Mark (X) one or more boxes.
[ ] White
[ ] Black, African Am., or Negro
[ ] American Indian or Alaska Native -- Print name of enrolled or principal tribe. -->
____
[ ] Asian Indian
[ ] Japanese
[ ] Chinese
[ ] Korean
[ ] Filipino
[ ] Vietnamese
[ ] Other Asian -- Print race, for example, Hmong, Laotian, Thai, Pakistani, Cambodian, and so on.____________________________
[ ] Native Hawaiian
[ ] Guamanian or Chamorro
[ ] Samoan
[ ] Other Pacific Islander -- Print race, for example, Fijian, Tongan, and so on.
[ ] Some other race -- Print race. -->
____

6. Mark all boxes for the appropriate races.
The concept of race, as used by the Census Bureau, reflects self-identification by individuals according to the race or races with which they identify.

© 2024 Ko M et al. *JAMA Network Open*.

The instruction before question 5, "For this survey, Hispanic origins are not races" reflects the federal government's treatment of Hispanic origin and race as separate and distinct concepts. People who identify their origin as Hispanic, Latino, or Spanish may be of any race.
People may choose to provide two or more races either by marking two or more race response boxes, by providing multiple write-in responses, or by some combination of marking boxes and writing in responses.
If you mark the "American Indian or Alaska Native" box, also print the name of the tribe(s) in which the person is enrolled. If the person is not enrolled in a tribe, print the name of the principal tribe.
If you mark the "Other Asian" box, print the name of the specific race(s) or group(s) in the space provided. The category "Other Asian" includes persons who identify themselves as Laotian, Thai, Pakistani, Cambodian, Sri Lankan, and so on.
If you mark the "Other Pacific Islander" box, print the name of the specific race(s) or group(s) in the space provided. The category "Other Pacific Islander" includes persons who identify themselves as Fijian, Tongan, Polynesian, Tahitian and so on.
If you mark the "Some other race" box, print the race(s) or group(s) in the space provided.
This question should be answered by ALL persons.

2014 - Race:
6. What is Person X's race? Mark (X) one or more boxes.
[ ] White
[ ] Black or African Am.
[ ] American Indian or Alaska Native -- Print name of enrolled or principal tribe. -->
____
[ ] Asian Indian
[ ] Japanese
[ ] Chinese
[ ] Korean
[ ] Filipino
[ ] Vietnamese
[ ] Other Asian -- Print race, for example, Hmong, Laotian, Thai, Pakistani, Cambodian, and so on.__________________________
[ ] Native Hawaiian
[ ] Guamanian or Chamorro
[ ] Samoan
[ ] Other Pacific Islander -- Print race, for example, Fijian, Tongan, and so on.
[ ] Some other race -- Print race. -->
____

6. Mark all boxes for the appropriate races.
The concept of race, as used by the Census Bureau, reflects self-identification by individuals according to the race or races with which they identify.
The instruction before question 5, "For this survey, Hispanic origins are not races" reflects the federal government's treatment of Hispanic origin and race as separate and distinct concepts. People who identify their origin as Hispanic, Latino, or Spanish may be of any race.

© 2024 Ko M et al. *JAMA Network Open*.

People may choose to provide two or more races either by marking two or more race response boxes, by providing multiple write-in responses, or by some combination of marking boxes and writing in responses.
If you mark the "American Indian or Alaska Native" box, also print the name of the tribe(s) in which the person is enrolled. If the person is not enrolled in a tribe, print the name of the principal tribe.
If you mark the "Other Asian" box, print the name of the specific race(s) or group(s) in the space provided. The category "Other Asian" includes persons who identify themselves as Laotian, Thai, Pakistani, Cambodian, Sri Lankan, and so on.
If you mark the "Other Pacific Islander" box, print the name of the specific race(s) or group(s) in the space provided. The category "Other Pacific Islander" includes persons who identify themselves as Fijian, Tongan, Polynesian, Tahitian and so on.
If you mark the "Some other race" box, print the race(s) or group(s) in the space provided.
This question should be answered by ALL persons.

2015 - Race:
6. What is Person X's race? Mark (X) one or more boxes.
[ ] White
[ ] Black or African Am.
[ ] American Indian or Alaska Native -- Print name of enrolled or principal tribe. -->
____
[ ] Asian Indian
[ ] Japanese
[ ] Chinese
[ ] Korean
[ ] Filipino
[ ] Vietnamese
[ ] Other Asian -- Print race, for example, Hmong, Laotian, Thai, Pakistani, Cambodian, and so on.__________________________
[ ] Native Hawaiian
[ ] Guamanian or Chamorro
[ ] Samoan
[ ] Other Pacific Islander -- Print race, for example, Fijian, Tongan, and so on.
[ ] Some other race -- Print race. -->
____

6. Mark all boxes for the appropriate races.
The concept of race, as used by the Census Bureau, reflects self-identification by individuals according to the race or races with which they identify.
The instruction before question 5, "For this survey, Hispanic origins are not races" reflects the federal government's treatment of Hispanic origin and race as separate and distinct concepts. People who identify their origin as Hispanic, Latino, or Spanish may be of any race.
People may choose to provide two or more races either by marking two or more race response boxes, by providing multiple write-in responses, or by some combination of marking boxes and writing in responses.

© 2024 Ko M et al. *JAMA Network Open*.

If you mark the "American Indian or Alaska Native" box, also print the name of the tribe(s) in which the person is enrolled. If the person is not enrolled in a tribe, print the name of the person's enrolled or principle tribe(s) in the space provided (for example, Navajo nation, Blackfeet Tribe, Muscogee (Creek) nation, Mayan, Doyon, native Village of Barrow Inupiat Traditional Government, and so on).
If you mark the "Other Asian" box, print the name of the specific Asian group(s) in the space provided (for example, Pakistani, Cambodian, Hmong, Laotian, Bangladeshi, and so on.)
If you mark the "Other Pacific Islander" box, print the name of the specific Pacific Islander group(s) in the space provided (for example, Tongan, Fijian, Marshallese, Palauan, Tahitian, Papua New Guinean, and so on.)
If you mark the "Some other race" box, print the race(s) or group(s) in the space provided.
This question should be answered by ALL persons.

2016 - Race:
6. What is Person 1's race? Mark (X) one or more boxes.
[ ] White
[ ] Black or African Am.
[ ] American Indian or Alaska Native -- Print name of enrolled or principal tribe. -->
____
[ ] Asian Indian
[ ] Japanese
[ ] Chinese
[ ] Korean
[ ] Filipino
[ ] Vietnamese
[ ] Other Asian -- Print race, for example, Hmong, Laotian, Thai, Pakistani, Cambodian, and so on.____________________________
[ ] Native Hawaiian
[ ] Guamanian or Chamorro
[ ] Samoan
[ ] Other Pacific Islander -- Print race, for example, Fijian, Tongan, and so on.
[ ] Some other race -- Print race. -->
____

6. Mark all boxes for the appropriate races.
The concept of race, as used by the Census Bureau, reflects self-identification by individuals according to the race or races with which they identify.
The instruction before question 5, "For this survey, Hispanic origins are not races" reflects the federal government's treatment of Hispanic origin and race as separate and distinct concepts. People who identify their origin as Hispanic, Latino, or Spanish may be of any race.
People may choose to provide two or more races either by marking two or more race response boxes, by providing multiple write-in responses, or by some combination of marking boxes and writing in responses.
If you mark the "American Indian or Alaska Native" box, print the name of the person's enrolled or principal tribe(s) in the space provided (for example, Navajo nation, Blackfeet Tribe,

© 2024 Ko M et al. *JAMA Network Open*.

Muscogee (Creek) nation, Mayan, Doyon, native Village of Barrow Inupiat Traditional Government, and so on).
If you mark the "Other Asian" box, print the name of the specific Asian group(s) in the space provided (for example, Pakistani, Cambodian, Hmong, Laotian, Bangladeshi, and so on.)
If you mark the "Other Pacific Islander" box, print the name of the specific Pacific Islander group(s) in the space provided (for example, Tongan, Fijian, Marshallese, Palauan, Tahitian, Papua New Guinean, and so on.)
If you mark the "Some other race" box, print the specific group(s) in the space provided.
This question should be answered by ALL persons.

2017 - Race:
6. What is Person 1's race? Mark (X) one or more boxes.
[ ] White
[ ] Black or African Am.
[ ] American Indian or Alaska Native -- Print name of enrolled or principal tribe. -->
____
[ ] Asian Indian
[ ] Japanese
[ ] Chinese
[ ] Korean
[ ] Filipino
[ ] Vietnamese
[ ] Other Asian -- Print race, for example, Hmong, Laotian, Thai, Pakistani, Cambodian, and so on.___________________________
[ ] Native Hawaiian
[ ] Guamanian or Chamorro
[ ] Samoan
[ ] Other Pacific Islander -- Print race, for example, Fijian, Tongan, and so on.
[ ] Some other race -- Print race. -->
____

2018 - Race:
6. What is Person 1's race? Mark (X) one or more boxes.
[ ] White
[ ] Black or African Am.
[ ] American Indian or Alaska Native -- Print name of enrolled or principal tribe. -->
____
[ ] Asian Indian
[ ] Japanese
[ ] Chinese
[ ] Korean
[ ] Filipino
[ ] Vietnamese
[ ] Other Asian -- Print race, for example, Hmong, Laotian, Thai, Pakistani, Cambodian, and so on.___________________________
[ ] Native Hawaiian

© 2024 Ko M et al. *JAMA Network Open.*

[ ] Guamanian or Chamorro
[ ] Samoan
[ ] Other Pacific Islander -- Print race, for example, Fijian, Tongan, and so on.
[ ] Some other race -- Print race. -->
____

2019 - Race:
6. What is Person X's race? Mark (X) one or more boxes.
[ ] White
[ ] Black or African Am.
[ ] American Indian or Alaska Native -- Print name of enrolled or principal tribe. -->
____
[ ] Asian Indian
[ ] Japanese
[ ] Native Hawaiian
[ ] Chinese
[ ] Korean
[ ] Guamanian or Chamorro
[ ] Filipino
[ ] Vietnamese
[ ] Samoan
[ ] Other Asian -- Print race, for example, Hmong, Laotian, Thai, Pakistani, Cambodian, and so on. --> ____________________
[ ] Other Pacific Islander ? Print race, for example, Fijian, Tongan, and so on. -->_____________________
[ ] Some other race -- Print race. -->
____

2020 - Race:
6. What is Person X's race? Mark (X) one or more boxes AND print origins.
[ ] White - Print, for example, German, Irish, English, Italian, Lebanese, Egyptian, etc. -->__________________________

[ ] Black or African Am. -Print, for example, African American, Jamaican, Haitian, Nigerian, Ethiopian, Somali, etc. -->________________________

[ ] American Indian or Alaska Native -- Print name of enrolled or principal tribe(s), for example, Navajo Nation, Blackfeet Tribe, Mayan, Aztec, Native Village of Barrow Inupiat Traditional Government, Nome Eskimo Community, etc. --> _____________________
[ ] Chinese
[ ] Vietnamese
[ ] Native Hawaiian
[ ] Filipino
[ ] Korean
[ ] Samoan
[ ] Asian Indian

[ ] Japanese
[ ] Chamorro
[ ] Other Asian -- Print, for example, Pakistani, Cambodian, Hmong, etc. -->
____
[ ] Other Pacific Islander ? Print, for example, Tongan, Fijian, Marshallese, etc. --
>_______________________
[ ] Some other race -- Print race or origin. -->
____

2021 - Race:
6. What is Person X's race? Mark (X) one or more boxesAND print origins.
[ ] White - Print, for example, German, Irish, English, Italian, Lebanese, Egyptian, etc. --
>____________________________

[ ] Black or African Am. -Print, for example, African American, Jamaican, Haitian, Nigerian, Ethiopian, Somali, etc. -->__________________________

[ ] American Indian or Alaska Native -- Print name of enrolled or principal tribe(s), for example, Navajo Nation, Blackfeet Tribe, Mayan, Aztec, Native Village of Barrow Inupiat Traditional Government, Nome Eskimo Community, etc. --> _______________________
[ ] Chinese
[ ] Vietnamese
[ ] Native Hawaiian
[ ] Filipino
[ ] Korean
[ ] Samoan
[ ] Asian Indian
[ ] Japanese
[ ] Chamorro
[ ] Other Asian -- Print, for example, Pakistani, Cambodian, Hmong, etc. -->
____
[ ] Other Pacific Islander ? Print, for example, Tongan, Fijian, Marshallese, etc. --
>_______________________
[ ] Some other race -- Print race or origin. -->
____

2022 - Race:
6. What is Person X's race? Mark (X) one or more boxesAND print origins.
[ ] White - Print, for example, German, Irish, English, Italian, Lebanese, Egyptian, etc. --
>____________________________

[ ] Black or African Am. -Print, for example, African American, Jamaican, Haitian, Nigerian, Ethiopian, Somali, etc. -->__________________________

© 2024 Ko M et al. *JAMA Network Open*.

[ ] American Indian or Alaska Native -- Print name of enrolled or principal tribe(s), for example, Navajo Nation, Blackfeet Tribe, Mayan, Aztec, Native Village of Barrow Inupiat Traditional Government, Nome Eskimo Community, etc. --> ______________________
[ ] Chinese
[ ] Vietnamese
[ ] Native Hawaiian
[ ] Filipino
[ ] Korean
[ ] Samoan
[ ] Asian Indian
[ ] Japanese
[ ] Chamorro
[ ] Other Asian -- Print, for example, Pakistani, Cambodian, Hmong, etc. -->
____
[ ] Other Pacific Islander ? Print, for example, Tongan, Fijian, Marshallese, etc. -->______________________
[ ] Some other race -- Print race or origin. -->
____

2007 - Occupation:
45. What kind of work was this person doing? (For example: registered nurse, personal manager, supervisor of order department, secretary, accountant)
____

45.Print one or more words to describe the kind of work the person did. If the person was a trainee, apprentice, or helper, include that in the description.
Enter descriptions like the following: registered nurse, personnel manager, supervisor of order department, secretary, accountant, high school teacher, etc.
Do not enter single words such as: nurse, manager, teacher, etc.

2008 - Occupation:
45. What kind of work was this person doing? (For example: registered nurse, personal manager, supervisor of order department, secretary, accountant)
____

45. Print one or more words to describe the kind of work the person did. If the person was a trainee, apprentice, or helper, include that in the description.
Enter descriptions like the following: registered nurse, personnel manager, supervisor of order department, secretary, accountant, high school teacher, etc.
Do not enter single words such as: nurse, manager, teacher, etc.

2009 - Occupation:
45. What kind of work was this person doing? (For example: registered nurse, personal manager, supervisor of order department, secretary, accountant)
____

© 2024 Ko M et al. *JAMA Network Open*.

45.Print one or more words to describe the kind of work the person did. If the person was a trainee, apprentice, or helper, include that in the description. Enter descriptions like the following: registered nurse, personnel manager, supervisor of order department, secretary, accountant, high school teacher, etc. Do not enter single words such as: nurse, manager, teacher, etc.

2010 - Occupation:
45. What kind of work was this person doing? (For example: registered nurse, personal manager, supervisor of order department, secretary, accountant)

____

45.Print one or more words to describe the kind of work the person did. If the person was a trainee, apprentice, or helper, include that in the description. Enter descriptions like the following: registered nurse, personnel manager, supervisor of order department, secretary, accountant, high school teacher, etc. Do not enter single words such as: nurse, manager, teacher, etc.

2011 - Occupation:
45. What kind of work was this person doing? (For example: registered nurse, personal manager, supervisor of order department, secretary, accountant)

____

45.Print one or more words to describe the kind of work the person did. If the person was a trainee, apprentice, or helper, include that in the description. Enter descriptions like the following: registered nurse, personnel manager, supervisor of order department, secretary, accountant, high school teacher, etc. Do not enter single words such as: nurse, manager, teacher, etc.

2012 - Occupation:
45. What kind of work was this person doing? (For example: registered nurse, personal manager, supervisor of order department, secretary, accountant)

____

45.Print one or more words to describe the kind of work the person did. If the person was a trainee, apprentice, or helper, include that in the description. Enter descriptions like the following: registered nurse, personnel manager, supervisor of order department, secretary, accountant, high school teacher, etc. Do not enter single words such as: nurse, manager, teacher, etc.

2013 - Occupation:
45. What kind of work was this person doing? (For example: registered nurse, personal manager, supervisor of order department, secretary, accountant)

____

45.Print one or more words to describe the kind of work the person did. If the

© 2024 Ko M et al. *JAMA Network Open*.

person was a trainee, apprentice, or helper, include that in the description.
Enter descriptions like the following: registered nurse, personnel manager, supervisor of order department, secretary, accountant, high school teacher, etc.
Do not enter single words such as: nurse, manager, teacher, etc.

2014 - Occupation:
45. What kind of work was this person doing? (For example: registered nurse, personal manager, supervisor of order department, secretary, accountant)
____

45.Print one or more words to describe the kind of work the person did. If the person was a trainee, apprentice, or helper, include that in the description.
Enter descriptions like the following: registered nurse, personnel manager, supervisor of order department, secretary, accountant, high school teacher, etc.
Do not enter single words such as: nurse, manager, teacher, etc.

2015 - Occupation:
45. What kind of work was this person doing? (For example: registered nurse, personal manager, supervisor of order department, secretary, accountant)
____

45.Print one or more words to describe the kind of work the person did. If the person was a trainee, apprentice, or helper, include that in the description.
Enter descriptions like the following: registered nurse, personnel manager, supervisor of order department, secretary, accountant, high school teacher, etc.
Do not enter single words such as: nurse, manager, teacher, etc.

2016 - Occupation:
45. What kind of work was this person doing? (For example: registered nurse, personal manager, supervisor of order department, secretary, accountant)
____

45. Describe the kind of work the person did. If the person was a trainee, apprentice, or helper, include that in the description.
Enter descriptions like the following: registered nurse, personnel manager, supervisor of order department, secretary, accountant, high school teacher, etc.
If possible, avoid single words such as: nurse, manager, and teacher.

2017 - Occupation:
45. What kind of work was this person doing? (For example: registered nurse, personal manager, supervisor of order department, secretary, accountant)
____

2018 - Occupation:
45. What kind of work was this person doing? (For example: registered nurse, personal manager, supervisor of order department, secretary, accountant)
____

© 2024 Ko M et al. *JAMA Network Open*.

2019 - Occupation:
45. What kind of work was this person doing? (For example: registered nurse, personal manager, supervisor of order department, secretary, accountant)

____

2020 - Occupation:
45. What kind of work was this person doing? (For example: registered nurse, personal manager, supervisor of order department, secretary, accountant)

____

2021 - Occupation:
45. What kind of work was this person doing? (For example: registered nurse, personal manager, supervisor of order department, secretary, accountant)

____

2022 - Occupation:
45. What kind of work was this person doing? (For example: registered nurse, personal manager, supervisor of order department, secretary, accountant)

____

© 2024 Ko M et al. *JAMA Network Open*.
