# Supplementary material for: Asian American Diversity and Representation in the Health Care Workforce, 2007 to 2022
Source: JAMA Netw Open. 2024 Oct 17;7(10):e2440071. doi: 10.1001/jamanetworkopen.2024.40071 (PMC11581573; doi:10.1001/jamanetworkopen.2024.40071)
Supplement: Supplement 2. — Data Sharing Statement [file jamanetwopen-e2440071-s002.pdf]

Data Sharing Statement

Ko. Asian American Diversity and Representation in the Health Care Workforce, 2007 to 2022. *JAMA Netw Open*. Published October 17, 2024. doi:10.1001/jamanetworkopen.2024.40071

Data
Data available: Yes
Data types: Data (not involving human participants), Data dictionary
How to access data: Data requests and dictionary can be addressed to: mijko@ucdavis.edu
When available:

Supporting Documents
Document types: None
